# Development of a quantitative index system for evaluating the quality of electronic medical records in disease risk intelligent prediction

**DOI:** 10.1186/s12911-024-02533-z

**Published:** 2024-06-24

**Authors:** Jiayin Zhou, Jie Hao, Mingkun Tang, Haixia Sun, Jiayang Wang, Jiao Li, Qing Qian

**Affiliations:** 1 Institute of Medical Information, Chinese Academy of Medical Sciences & Peking Union Medical College, Beijing, People’s Republic of China; 2 Chinese Academy of Medical Sciences & Peking Union Medical College, Beijing, People’s Republic of China

**Keywords:** Electronic medical record, Quality control, Data management, Machine learning, Disease risk prediction

## Abstract

**Objective:**

This study aimed to develop and validate a quantitative index system for evaluating the data quality of Electronic Medical Records (EMR) in disease risk prediction using Machine Learning (ML).

**Materials and methods:**

The index system was developed in four steps: (1) a preliminary index system was outlined based on literature review; (2) we utilized the Delphi method to structure the indicators at all levels; (3) the weights of these indicators were determined using the Analytic Hierarchy Process (AHP) method; and (4) the developed index system was empirically validated using real-world EMR data in a ML-based disease risk prediction task.

**Results:**

The synthesis of review findings and the expert consultations led to the formulation of a three-level index system with four first-level, 11 second-level, and 33 third-level indicators. The weights of these indicators were obtained through the AHP method. Results from the empirical analysis illustrated a positive relationship between the scores assigned by the proposed index system and the predictive performances of the datasets.

**Discussion:**

The proposed index system for evaluating EMR data quality is grounded in extensive literature analysis and expert consultation. Moreover, the system’s high reliability and suitability has been affirmed through empirical validation.

**Conclusion:**

The novel index system offers a robust framework for assessing the quality and suitability of EMR data in ML-based disease risk predictions. It can serve as a guide in building EMR databases, improving EMR data quality control, and generating reliable real-world evidence.

**Supplementary Information:**

The online version contains supplementary material available at 10.1186/s12911-024-02533-z.

## Introduction

The onset of the digital health era has led to a paradigm shift in health management, transitioning from a focus on reactive treatment to proactive prevention [1]. Disease risk intelligent prediction has become a vital strategy in proactive health management, aiming to identify potential risk factors and prevent the progression of diseases. By harnessing the capabilities of Artificial Intelligence (AI) technologies and Machine Learning (ML) approaches, healthcare professionals can gain valuable insights into diseases, enabling the development of more effective preventive treatment plans [2, 3].

Johnson [4] applied four different ML-based models to predict subsequent deaths or cardiovascular events in a cohort of 6,892 patients. The study found that the ML-based model had superior discrimination ability compared to traditional coronary Computed Tomography (CT) scores in identifying patients at risk of adverse cardiovascular events. Electronic medical records (EMR) data, as a valuable real-world data source, plays a critical role in disease risk prediction using ML techniques [4]. An EMR refers to a digital version of a patient’s medical record, encompassing medical history, medications, test results, and other relevant information [5, 6]. Healthcare providers commonly utilize EMRs to document and track patient information, enabling comprehensive decision-making regarding patient care. Furthermore, clinical researchers can leverage de-identified EMR data to study disease patterns, develop novel treatments, and advance medical knowledge. The integration of ML with EMRs has recently shown significant improvements in predicting patient outcomes, such as identifying individuals with suspected coronary artery disease [7] or forecasting the likelihood of open-heart surgery [8]. These advancements highlight the potential of ML in enhancing the efficiency of clinical decision-making [9].

Nevertheless, several studies have raised concerns about the quality of EMRs in clinical research, emphasizing issues such as lack of data standardization, incomplete or missing clinical data, and discrepancies in data types and element representations [10, 11]. Ensuring the quality of EMR data is crucial, as it forms the bedrock for effective utilization of EMRs. High-quality EMR data not only supplies robust evidence, but also accelerates the clinical research process, shortens its timeline, and reduces associated risks. Therefore, controlling and evaluating EMR data quality are pivotal in upholding the overall quality and integrity of clinical research.

Despite numerous studies investigating the assessment of EMR data quality in clinical research, it is noteworthy that the body of literature evaluating EMR data quality is growing [12, 13]. However, publicly published clinical studies employing ML techniques and utilizing EMR data frequently overlook data quality or implement methods lacking expert knowledge or evidential support. While methods for data quality evaluation have been described in the informatics literature, researchers without specialized knowledge in this field may find difficulty choosing the appropriate evaluation method in line with the available data and research problems [14]. Furthermore, the existing quality assessment framework primarily relies on qualitative approaches, making objective measurement of quality and suability challenging.

In this paper, we aim to develop and validate a quantitative index system for evaluating the quality of EMR in disease risk prediction using ML. The proposed index system is intended to provide guidance for utilizing EMR data in research, enhance the quality of EMR data within a Hospital Information System (HIS), and facilitate the implementation of clinical decision-making research based on EMR data. By applying the proposed index system, researchers and healthcare professionals can make knowledgeable decisions regarding the use of EMR data for ML-based disease prediction research, ultimately improving patient care and advancing medical knowledge.

## Materials and methods

**Fig. 1:**
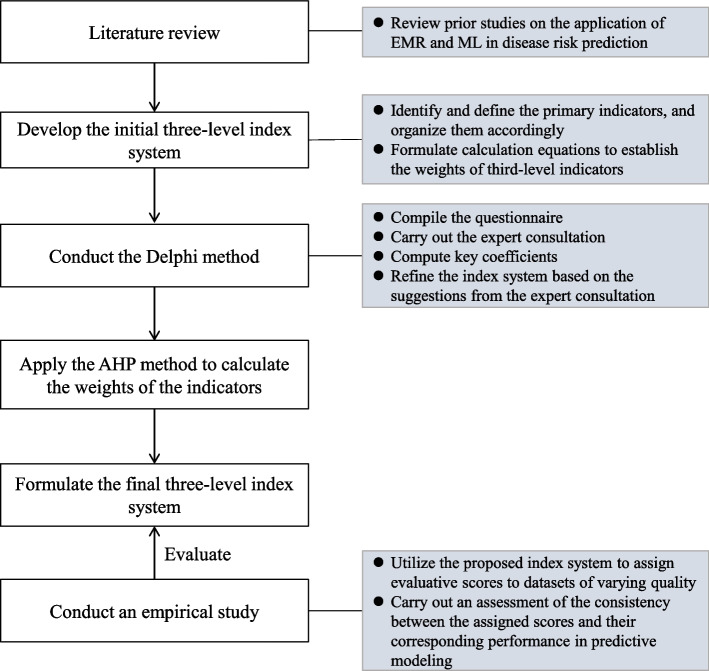
Workflow of the study

In this paper, we present the development of a quantitative index system, depicted in Fig. 1, designed to ensure the quality control of EMR data in disease prediction models. The development process incorporated the use of the Delphi method and the analytic hierarchy process (AHP). In addition, an empirical study was undertaken to validate the effectiveness of the developed index system using real-world EMR data in disease risk intelligent prediction.

### Sketching the preliminary index system

#### Preliminary indicator identification, definition, and organization

The initial set of indicators was determined through a comprehensive literature review of studies published before September 27, 2021, obtained from the PubMed database. The search query used was “(machine learning) AND (electronic medical records) AND (disease prediction)”, which resulted in 549 papers. The inclusion criteria required that the research data be related to EMR or HIS and that disease risk was predicted using ML techniques. Review articles and papers deemed to have low relevance were excluded, leading to the removal of 225 papers based on the fulfillment of the exclusion criteria after reading abstracts.

**Fig. 2:**
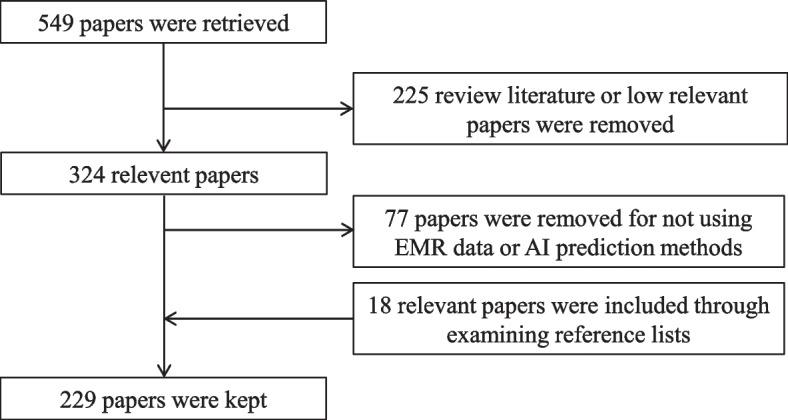
Flowchart of paper screening

Further screening was conducted by reading the full papers to eliminate studies that did not involve EMR or HIS data or utilized disease prediction methods other than machine learning. Additionally, 18 relevant papers were included by examining the reference lists of the selected studies. Ultimately, a total of 229 papers were retained for the development of the preliminary index system. The detailed process of paper screening is illustrated in Fig. 2.

Upon analyzing the review results, we formulated an initial multi-level index system consisting of four first-level, 11 second-level, and 33 third-level indicators. The first-level indicators represent broad dimensions of data quality, while the second-level indicators correspond to the general dimensions specifically for EMR data quality. The third-level indicators capture specific dimensions relevant to EMR-based disease prediction models.

#### Calculation methods

We utilized the AHP method to determine the weights of the first- and second-level indicators in the three-level index system. The weights of the third-level indicators were calculated using percentages or binary values according to their definitions. The calculation formulas of these third-level indicators will be assessed in the forthcoming Delphi consultation.

### Developing a three-level index system using the Delphi method

#### Questionnaire compilation and expert consultation

We conducted a Delphi consultation to gather feedback from experts based on the preliminary index system. The consultation questionnaire, provided in Additional file 1, consists of four parts: experts' basic information (see Table S1), familiarity and judgment basis with AI-based disease prediction (see Table S2-S3), evaluation tables for the preliminary index system (see Table S4-S6), and an evaluation table for the calculation formulas of the third-level indicators (see Table S7). The importance of the preliminary indicators was measured using a 5-point Likert scale, ranging from “very unimportant” to “very important”. To ensure the extensibility of the preliminary index system, three additional options were included: delete, modify, and new indicator(s). For the calculation formulas part, experts were asked to provide a yes or no response, and if the answer was no, a suggestion for modification was requested.

A total of twenty experts specializing in healthcare/EMR data governance and medical AI were selected for the Delphi consultation. The inclusion criteria for the selection were as follows: (1) holding a Ph.D. degree or being a senior technical associate; (2) possessing more than two years of research experience in related fields; (3) being familiar with the construction and evaluation of EMR data; and (4) being able to give feedback in a timely manner. We conducted a single-round consultation since the nature of our consulting panel was relatively small and homogeneous [15].

#### Key coefficients and statistical analyses

To achieve relatively consistent and reliable feedback from the questionnaire, we calculated four metrics: the experts' positive coefficients, expert authority coefficients (Cr), coefficient of variation (CV), and Kendall's coefficient of concordance. The experts' positive coefficients were determined based on the response rate to the questionnaire. A response rate of 70% or higher is considered satisfactory [16]. The Cr was calculated as the average of the familiarity coefficient (Cs) and the judgment coefficient (Ca), reflecting the reliability of the expert consultation. A Cr value of 0.7 or above is considered acceptable. The CV measures the consistency of indicators on the same level. A CV value less than 0.25 is expected, indicating a high level of consistency [17]. Kendall's coefficient of concordance evaluates the overall consistency of all indicators in the system. It ranges from zero to one, with a value greater than 0.2 considered acceptable [18]. All statistical analyses were performed using Microsoft Excel/IBM SPSS 25.0.

### Using the AHP method for weight assignment

We applied the AHP method to determine the weights of indicators at each level, which is a well-known technique in multiple criteria decision-making [19]. AHP enables the quantification of criteria and opinions that are difficult to measure numerically, and its outcomes are free from subjective influence due to its use of pairwise comparisons and eigenvalues [20].

In this study, our AHP method was conducted in three steps. First, we obtained the importance ratings of experts for each indicator. Then, we averaged these ratings for each indicator and performed pairwise comparisons among indicators at the same level that belong to the same upper level. This step allowed us to construct multiple judgment matrices based on their ratios.

Second, we calculated the eigenvectors of each indicator by normalizing the judgement matrix. A larger eigenvector for an indicator represents a higher relative importance. The relative weights of indicators at the same level were determined by standardizing the eigenvectors. For the first-level indicators, their relative weights were equal to their absolute weights. For the second- and third-level indicators, their absolute weights were calculated by multiplying their relative weights with the absolute weight of the upper level.

**Fig. 3:**
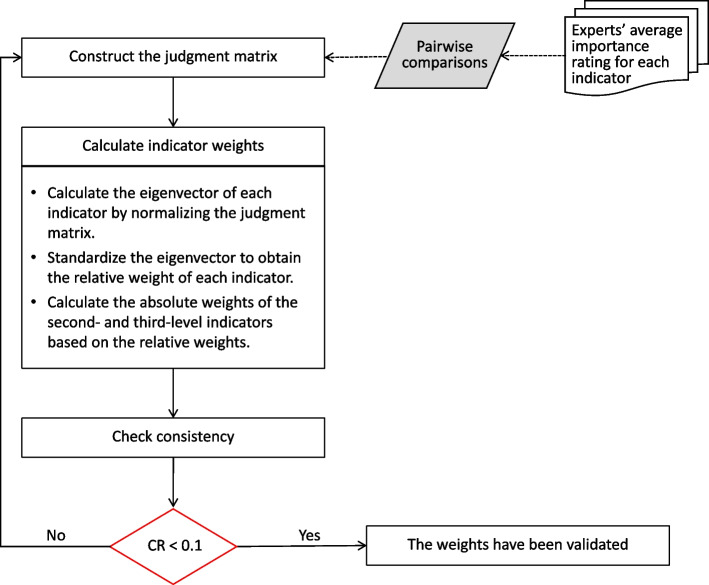
Flowchart of the AHP method

Third, we performed a consistency test using the consistency ratio (CR) to evaluate the consistency of the judgment matrices. A CR below 0.1 indicated that the judgment matrices were consistent and that the obtained weights were considered valid [21]. The steps of the AHP method are illustrated in Fig. 3.

### Evaluating the index system through prediction tasks

To further validate the suitability of the proposed index system, an empirical study was conducted using real-world EMR data for disease risk prediction.

#### Dataset construction

To ensure a fair assessment, we opted to generate multiple datasets from a single EMR data resource. The chosen data resource needed to be large-scale, open-access, and regularly updated. Once the data resource was identified, we constructed several datasets with varying sample types but maintaining the same set of attributes.

For each dataset, we computed the scores of 33 third-level indicators using the established calculation formulas. The weights of the proposed index system were applied to obtain weighted scores for all indicators within each dataset. The overall score of a dataset was subsequently computed by summing the scores of the first-level indicators.

#### Predictive modeling

In the context of disease risk prediction, we considered three widely used ML models: logistic regression (LR), support vector machine (SVM), and random forest (RF). LR is a traditional classification algorithm used to estimate the probability of an event occurring [22]. SVM, a nonlinear classifier, employs a kernel function to transform input data into a higher-dimensional space, making it effective in handling complex relationships and nonlinear patterns [23]. RF is an ensemble method that combines the predictions from multiple decision trees. It has shown great success in disease risk prediction tasks by reducing overfitting and improving predictive accuracy [24]. For our analysis, we used the scikit-learn python library [25] to implement LR, SVM, and RF.

#### Reliability analysis

In our study, we conducted reliability analysis to examine the relationship between the scores obtained from our constructed datasets and the performance of predictive models. we applied Pearson correlation for assessing linear relationships [26] and Spearman correlation for nonlinearity [27]. The Pearson correlation coefficient was calculated using the formula:

1 $$r=\frac{{\sum }_{i=1}^{n}({x}_{i}-\overline{x })({y}_{i}-\overline{y })}{\sqrt{{\sum }_{i=1}^{n}({x}_{i}-{\overline{x })}^{2}{\sum }_{i=1}^{n}({y}_{i}-\overline{y }{)}^{2}}}$$

Here, ${x}_{i}$ and ${y}_{i}$ represent individual data points from the two respective datasets, while $\overline{x }$ and $\overline{{\text{y}} }$ denote the mean values of these datasets. A Pearson correlation coefficient near 1 or -1 indicates a strong linear relationship between dataset scores and model performance, whereas a value close to 0 suggests a very weak linear relationship.

Similarly, the Spearman correlation coefficient was calculated using the formula:

2 $$\rho =1-\frac{6\sum {d}_{i}^{2}}{n\left({n}^{2}-1\right)}$$

Here, ${d}_{i}$ represents the difference in rank between the two datasets for the *i*-th observation, and *n* denotes the total number of observations. A Spearman correlation coefficient near 1 or -1 indicates a strong nonlinear relationship, while a value close to 0 suggests a very weak relationship.

In both analyses, statistical significance was established with a p-value less than 0.05. This finding indicates a significant correlation between the scores of our constructed datasets and the performance of predictive models. Thus, this statistically significant outcome supports the reliability of our proposed index system in evaluating the data quality of EMR for intelligent disease risk prediction.

## Results

### The characteristics of the experts

In the Delphi consultation, a total of twenty experts were invited, of which 17 actively participated, yielding a response rate of 85.0%. Out of the 17 experts, 16 provided feedback that met the credibility criteria for a Delphi study, resulting in an effective response rate of 94.1%. These response rates reflect a high degree of expert engagement.

Most of the participating experts were male, held Ph.D. degrees, and specialized in medical informatics or medical AI. Over half of the experts were aged between 40 and 50 years, and 62.5% had between 10 and 20 years of work experience. Moreover, 68.7% of the experts occupied senior associate positions or higher. For detailed information, see Table S8 in Additional file 3.

### The key coefficients of the Delphi method

The degree of expert authority (Cr) is defined by two factors: the expert's familiarity with the consultation content (Cs) and the basis of expert judgment (Ca). Of the 16 participating experts, 7 were found to be very familiar with the content, while 9 were relatively familiar. This indicates an overall sound understanding of the field among the experts. Only two experts exhibited a low judgment basis, suggesting that the majority of the experts were well-equipped to offer informed judgment. Details of expert familiarity and judgment basis can be found in Table S9 in Additional file 3.

Cr was calculated to be 0.89, with Cs and Ca values of 0.88 and 0.90, respectively. These values indicate a high level of expert authority and reliability in the consultation results. The CV values for the first-level indicators were less than 0.16, for the second-level indicators were less than 0.20, and for the third-level indicators were no more than 0.25. These low CV values indicate a high level of consistency among experts' scores for the preliminary indicators at each level. Kendall's coefficients of concordance for all three levels were greater than 0.30, indicating a substantial level of agreement among the experts. Additionally, the p-values for the preliminary second- and third-level indicators were very small, further confirming the consistency of experts' scores for each preliminary indicator. Overall, the results demonstrate a high level of consistency and reliability in the experts' assessments for each preliminary indicator.

### The final weighted three-level index system

Experts' comments focused on changes to the definition of indicators. After further discussions with experts, all preliminary indicators were included in the final weighted three-level index system, as shown in Table 1. No new indicators were added to the system. The index system comprises four first-level indicators, 11 second-level indicators, and 33 third-level indicators, with the weights determined using the AHP method and percentages.

**Table 1:** The final weighted three-level index system

First-Level Indicators name (weight)	Second-Level Indicators name (weight)	Third-Level Indicators name (weight)
Operability (0.251)	Integrability (0.091)	Ratio of mapping the primary key (0.032)
		Ratio of mapping data elements (0.030)
		Ratio of interconvertible data elements (0.029)
	Portability (0.082)	Reliable data migration (0.082)
	Selectivity (0.078)	Sufficient data elements as inputs for feature engineering (0.020)
		Sufficient data elements as outputs for feature engineering (0.019)
		Measurement selectivity in data inputs for feature engineering (0.020)
		Measurement selectivity in data outputs for feature engineering (0.019)
Completeness (0.254)	Integrity of data elements (0.066)	Integrity of data elements as inputs in predictive modeling (0.017)
		Integrity of data elements as outputs in predictive modeling (0.016)
		Integrity of data values as inputs in predictive modeling (0.017)
		Integrity of data values as outputs in predictive modeling (0.016)
	Integrity of temporal information (0.062)	Integrity of timestamps as creating data (0.032)
		Integrity of timestamps as creating values with data (0.031)
	Integrity of data state (0.063)	Integrity of data state in available (0.063)
	Data balance (0.063)	Adequate data (0.020)
		Balance of input data (0.021)
		Balance of output data (0.022)
Correctness (0.264)	Data accuracy (0.087)	Accurate data formats (0.013)
		Accurate data types (0.015)
		Right level of granularity (0.015)
		Accurate measurement of data (0.015)
		Unambiguity of data elements (0.015)
		Unambiguity of measurement of data (0.014)
	Data consistency (0.089)	Consistent measurement of data (0.031)
		Consistent metric calculations (0.030)
		Consistent metric units (0.028)
	Data compliance (0.089)	Data elements for compliance (0.023)
		Data measurement for compliance (0.022)
		Standard timestamps for compliance (0.022)
		Standard time logs for compliance (0.023)
Timeliness (0.231)	Data timeliness (0.231)	Timeliness on recording data (0.113)
		Frequency on recording data (0.118)

The first-level indicators represent a series of data quality characteristics that determine the suitability of EMR data for disease risk intelligent prediction research. The second-level indicators provide a concrete representation or evaluation of the first-level indicators, making it easier for users to understand their extension or evaluation. The third-level indicators further specify the second-level indicators, providing clear quality requirements for different levels of granularity in the EMR dataset, such as data records, data elements, and data element values. This facilitates users in understanding the evaluation needs and contents more clearly. For detailed information on the indicators, please see Additional file 2.

### Data preprocessing

In this empirical study, the MIMIC-III clinical database was chosen as the representative real-world EMR data resource. MIMIC-III^1^ is an extensive and freely accessible database that contains comprehensive health-related data from more than 46,000 patients admitted to intensive care unit (ICU) at the Beth Israel Deaconess Medical Center between 2001 and 2012 [28]. For this study, we utilized MIMIC-III v1.4, which is the latest version released in 2016 [29] and ensures effective control over EMR data.

Sepsis is a leading cause of mortality among ICU patients, highlighting the importance of accurate sepsis risk prediction for precise treatments in the ICU [30]. Hence, we selected sepsis as the disease prediction task using the MIMIC-III database. Potential predictors were extracted from the records of vital signs, routine blood examinations [31], liver function tests [32] and demographic information. The outcome variable for the prediction task is the occurrence of sepsis. Furthermore, we obtained five different populations of ICU patients with a high risk of sepsis from the MIMIC-III database. The number of patients in each population, categorized as elderly (> 80 years old), long-stay (> 30 days of length of stay, LLOS), ischemic stroke, acute renal failure (ARF), and cirrhosis (CIR), is presented in Table S10 in Additional file 3.

### Scoring datasets

According to the proposed index system, we evaluated the five datasets and assigned scores to each indicator based on their respective weights in the system. The detailed list of scores of divergent indicators for each dataset can be found in Table S11 in Additional file 3. In Table 2, we present the scores of first-level indicators. It is important to note that the scores for the operability indicator were consistent across all five datasets, with a value of 0.251. This is because these datasets were obtained from a single resource.

**Table 2:** Overall scores of the five datasets

Dataset	Results				
	Operability (0.251)	Completeness (0.254)	Correctness (0.264)	Timeliness (0.231)	Total Score (Up to 1)
Elderly	**0.251**	**0.250**	0.260	0.174	0.935
LLOS	**0.251**	0.227	**0.264**	**0.224**	**0.966**
Stroke	**0.251**	**0.250**	0.263	0.177	0.941
ARF	**0.251**	0.230	0.263	0.163	0.907
CIR	**0.251**	0.231	**0.264**	0.183	0.929

When considering the overall scores, the LLOS dataset achieved the highest score of 0.966, indicating a higher level of quality, while the ARF dataset obtained the lowest score of 0.907. These scores provide an assessment of the datasets' suitability and quality for disease risk prediction using the proposed index system.

### Experimental results

Additional data processing was conducted to prepare the datasets for training ML models. To address the missing values, median imputation was applied to predictors with a small proportion of missing values in each dataset. To mitigate potential bias arising from imbalanced datasets, we applied undersampling on the majority class to achieve a balanced ratio of 1:1. Each dataset was then randomly split into 80% for training and 20% for testing. To ensure fairness in model comparison, the predictors were normalized, and a tenfold cross-validation was performed during the training process.

Regarding model hyperparameters, the LR model applied the 'liblinear' solver method. The SVM model utilized a Radial Basis Function kernel, with a regularization parameter (C) set to 1.0, and the gamma value was set to 'scale'. For the RF model, it was constructed with 10 trees (n_estimators = 10), a maximum tree depth of 7 (max_depth = 7), and optimal feature selection (max_features = ‘auto’).

The evaluation of model performance was based on accuracy (ACC), precision, and area under the curve (AUC). Accuracy represents the proportion of correct predictions made by a model among all predictions. Precision measures the proportion of true positive predictions among all positive predictions made by a model. AUC, also known as the area under the receiver operating characteristic curve, is a metric used to evaluate the performance of binary classification models [33].

Table 3 displays the model performance on the five datasets. Among the three models, LLOS achieved the highest performance across all three evaluation metrics. On the other hand, ARF had the lowest performance in most cases, except for precision in the LR model.

**Table 3:** Model performance on the five datasets

Dataset	RF			SVM			LR		
	ACC	Precision	AUC	ACC	Precision	AUC	ACC	Precision	AUC
Elderly	0.699	0.671	0.645	0.712	0.661	0.650	0.698	0.647	0.634
LLOS	**0.858**	**0.927**	**0.855**	**0.869**	**0.908**	**0.855**	**0.884**	**0.935**	**0.866**
Stroke	0.805	0.730	0.703	0.829	0.735	0.716	0.800	0.625	0.666
ARF	0.630	0.646	0.619	0.559	0.622	0.547	0.606	0.694	0.596
CIR	0.767	0.800	0.737	0.837	0.688	0.737	0.767	0.733	0.670

### Association analysis

The relationships between the scores of datasets and the performance metrics of the models were analyzed as follows. First, a normality test was conducted on each pair of scores. If the scores passed the normality test, a Pearson correlation analysis was performed. Otherwise, a Spearman correlation analysis was conducted. Table 4 shows that all correlations, except for LR-Precision, were strongly positive and statistically significant. The SVM-Precision correlation showed the strongest effect among them.

**Table 4:** Correlation results of each dataset

Combination	Correlation Coefficient	*P*-value
RF-Accuracy	0.907^*^	0.017
RF-Precision	0.825^*^	0.043
RF-AUC	0.866^*^	0.029
SVM-Accuracy	0.819^*^	0.045
SVM-Precision	0.933^*^	0.010
SVM-AUC	0.924^*^	0.012
LR-Accuracy	0.932^*^	0.010
LR-Precision	0.639	0.123
LR-AUC	0.905^*^	0.017

^*^Correlation is significant at the 0.05 level (right-tailed)

## Discussion

We have developed a quantitative evaluation index system to assess the suitability of EMRs in disease risk intelligent prediction research. The proposed index system was validated through an empirical study using MIMIC-III datasets for predicting sepsis. Three popular ML models were performed, and the predictive results demonstrated that datasets with higher scores achieved better performance across three ML models. Our result is consistent with a previous study that showed the impact of data quality on prediction performance [34]. Additionally, the association analyses revealed a strong positive relationship between the scores of datasets and the combination of the ML model and evaluation metric. These findings confirm that the proposed index system was effective in evaluating the quality of EMR data in disease risk prediction using ML techniques.

Compared to the general framework for evaluating EMR data quality, our proposed index system was constructed by incorporating both the quality characteristics of EMR data and the specific research activities in ML-based disease risk prediction. It differs from the frameworks developed by Johnson [35] and Lv [36], which focused on summarizing literature on general medical data rather than specifically on EMR data. Although Weiskopf [37] specified EMR data as a required condition for a literature search, they did not explicitly address the situation of using EMR data in their development. The proposed index system considers not only the practical foundation of EMR data but also the data processing operations and operational objectives of EMRs at different stages of prediction model construction. This approach makes the evaluation index system more focused on its research purpose and enhances its explanatory power.

Another significant contribution of the proposed index system is the quantitative evaluation of EMR data quality in disease risk prediction. This provides researchers with guidance or standards for quantifying the EMR datasets for specific research purposes. Most current EMR data quality evaluation systems for clinical research rely on qualitative indicators [38]. Qualitative indicators are often based on typical cases, statements, and supporting materials, which may lack objectivity. While the study of Weiskopf [37] incorporated quantitative evaluation, it still relied on subjective scoring of each dimension by experts to calculate the mean value. The evaluation model proposed by Zan [39] utilized objective measurement indicators, but it primarily focused on binary classification and only included first-level indicators.

The proposed three-level index system was developed using a combination of qualitative and quantitative approaches. The naming and definition of all three levels of indicators were constructed through an extensive literature review and expert consultation. The first-level indicators correspond to the core qualitative aspects of evaluating EMR data quality in ML-based disease risk prediction. The second-level indicators serve as a refinement of the first-level qualitative indicators. The third-level indicators are quantitative in nature and can be obtained through objective quantitative calculations, such as assessing the coverage bias of the outcome variables in the integrity of the third-level indicators. Through the AHP method, the weights of the first- and second-level indicators can be obtained by the weights of third-level indicators in a hierarchical way.

Our study has several limitations. First, the calculation of the weights for the third-level indicators was based on simple percentages. This calculation method may neglect variations in the importance of different indicators. Second, the empirical study was conducted using the MIMIC-3 v1.4 database. Although the MIMIC-3 dataset is a widely used resource in research, the use of a single data resource may restrict the generalizability of our findings. Certain indicators for comparing data resources may be hard to validate without diverse EMR data resources. Hence, future validation studies using another data resource should be conducted to ensure the robustness of the proposed index system.

## Conclusion

In this paper, we developed a quantitative three-level index system, which included four first-level, 11 second-level, and 33 third-level indicators, to evaluate the EMR data quality in ML-based disease risk prediction. The reliability of the proposed index system has been verified through an empirical study with real-world data.

The proposed index system can benefit both EMR users for research and data managers. For EMR users for research, the proposed index system could provide them with a measurement for the suitability of EMR data in ML-based disease risk predictions. For EMR data managers, it could guide the direction of EMR database construction and improve the EMR data quality control. Eventually, we hope that the proposed index system can promote the generation of real-world evidence from reliable real-world EMR data.

### Supplementary Information

Supplementary Material 1.

Supplementary Material 2.

Supplementary Material 3. 

## Data Availability

All data generated or analyzed during this study are included in this published article and its additional files.

## Abbreviations

ACC: Accuracy
AI: Artificial intelligence
AHP: Analytic hierarchy process
ARF: Acute renal failure
AUC: Area under curve
Ca: Judgment coefficient
CIR: Cirrhosis
CR: Consistency ratio
Cr: Expert authority coefficients
Cs: Familiarity coefficient
CT: Computed tomography
CV: Coefficient of variation
EMR: Electronic medical record
HIS: Hospital information system
ICU: Intensive care units
LLOS: > 30 Days of length of stay
LR: Logistic regression
ML: Machine learning
SVM: Support vector machine
RF: Random forest

## Footnotes

1 https://physionet.org/content/mimiciii/1.4/

## References

[1] Waldman SA, Terzic A (2019). Healthcare evolves from reactive to proactive. Clin Pharmacol Ther.

[2] Razzak MI, Imran M, Xu G (2020). Big data analytics for preventive medicine. Neural Comput Appl.

[3] Davenport T, Kalakota R (2019). The potential for artificial intelligence in healthcare. Future Healthc J.

[4] Dzau VJ, Balatbat CA (2018). Health and societal implications of medical and technological advances. Sci Transl Med..

[5] Institute of Medicine (1997). The Computer-Based Patient Record: An Essential Technology for Health Care.

[6] Ambinder EP (2005). Electronic health records. Journal of oncology practice.

[7] Motwani M, Dey D, Berman DS (2017). Machine learning for prediction of all-cause mortality in patients with suspected coronary artery disease: a 5-year multi centre prospective registry analysis. Eur Heart J.

[8] Allyn J, Allou N, Augustin P (2017). A comparison of a machine learning model with EuroSCORE II in predicting mortality after elective cardiac surgery: a decision curve analysis. PLoS ONE.

[9] Geissbuhler A, Miller RA (1998). Clinical application of the UMLS in a computerized order entry and decision-support system. Proc AMIA Symp.

[10] Field D, Sansone SA (2006). A special issue on data standards. OMICS.

[11] Mead CN (2006). Data interchange standards in healthcare IT–computable semantic interoperability: now possible but still difficult, do we really need a better mousetrap?. J Healthc Inf Manag.

[12] Reimer AP, Milinovich A, Madigan EA (2016). Data quality assessment framework to assess electronic medical record data for use in research. Int J Med Inform.

[13] Johnson SG, Speedie S, Simon G (2016). Application of an ontology for characterizing data quality for a secondary use of EHR data. Appl Clin Inform.

[14] Ozonze O, Scott PJ, Hopgood AA (2023). Automating electronic health record data quality assessment. J Med Syst.

[15] Strasser A. Delphi method variants in IS research: a taxonomy proposal. In: PAC15 2016 Proceedings. 2016. https://aisel.aisnet.org/pacis2016/224. Accessed 5 July 2023.

[16] Babbie ER (2014). The Practice of Social Research.

[17] Bryman A (2015). Social Research Methods.

[18] Ruan Y, Song S, Yin Z (2022). Comprehensive evaluation of military training-induced fatigue among soldiers in China: A Delphi consensus study. Front Public Health.

[19] Shim JP (1989). Bibliographical research on the analytic hierarchy process (AHP). Socio-Econ Plann Sci.

[20] Ho W (2008). Integrated analytic hierarchy process and its applications–A literature review. Eur J Oper Res.

[21] Lane EF, Verdini WA (1989). A consistency test for AHP decision makers. Decis Sci.

[22] Johnson AE, Pollard TJ, Mark RG. “MIMIC-III clinical database (version 1.4),” PhysioNet. 2016; 10.13026/C2XW26.

[23] Nusinovici S, Tham YC, Yan MYC (2020). Logistic regression was as good as machine learning for predicting major chronic diseases. J Clin Epidemiol.

[24] Watanabe T, Kessler D, Scott C (2014). Disease prediction based on functional connectomes using a scalable and spatially-informed support vector machine. Neuroimage.

[25] Yang L, Wu H, Jin X (2020). Study of cardiovascular disease prediction model based on random forest in eastern China. Sci Rep.

[26] Pedregosa F, Varoquaux G, Gramfort A (2011). Scikit-learn: Machine learning in Python. J Machine Learn Res..

[27] Cohen I, Huang Y, Chen J, et al. Pearson correlation coefficient. In: Noise reduction in speech processing. Heidelberg: Springer; 2009:1–4.

[28] Hauke J, Kossowski T (2011). Comparison of values of Pearson's and Spearman's correlation coefficients on the same sets of data. Quaestion Geograph.

[29] Johnson AEW, Pollard TJ, Shen L (2016). MIMIC-III, a freely accessible critical care database. Scientific data.

[30] Mayr FB, Yende S, Angus DC (2014). Epidemiology of severe sepsis. Virulence.

[31] Lan P, Wang SJ, Shi QC (2018). Comparison of the predictive value of scoring systems on the prognosis of cirrhotic patients with suspected infection. Medicine.

[32] Lan P, Pan K, Wang S (2018). High serum iron level is associated with increased mortality in patients with sepsis. Sci Rep.

[33] Saito T, Rehmsmeier M (2017). Precrec: fast and accurate precision-recall and ROC curve calculations in R. Bioinformatics.

[34] Ferencek A, Kljajić BM (2020). Data quality assessment in product failure prediction models. J Decis Syst.

[35] Johnson SG, Speedie S, Simon G (2015). A data quality ontology for the secondary use of EHR data. AMIA Annu Symp Proc.

[36] Tian Q, Chen Y, Han Z (2020). Research on evaluation indexes of clinical data quality. J Med Inform.

[37] Weiskopf NG, Bakken S, Hripcsak G (2017). A data quality assessment guideline for electronic health record data reuse. EGEMS (Wash DC).

[38] Kahn MG, Callahan TJ, Barnard J (2016). A harmonized data quality assessment terminology and framework for the secondary use of electronic health record data. EGEMS (Wash DC).

[39] Cai L, Zhu Y (2015). The challenges of data quality and data quality assessment in the big data era. Data Sci J.

