# Supplementary material for: Development of a quantitative index system for evaluating the quality of electronic medical records in disease risk intelligent prediction
Source: BMC Med Inform Decis Mak. 2024 Jun 24;24:178. doi: 10.1186/s12911-024-02533-z (PMC11194906; doi:10.1186/s12911-024-02533-z)
Supplement: Supplementary file 1 — Supplementary Material 1. [file 12911_2024_2533_MOESM1_ESM.docx]

**Additional file 1**

Expert Consultation Questionnaire Form on the Research of Evaluation Index System for the Applicability of Electronic Medical Records

**Expert Consultation Questionnaire on the Construction of Evaluation Index System for the Applicability of Electronic Medical Record Data in Disease Risk Intelligent Prediction Research**

Dear experts:

Thank you so much for participating in the expert consultation!

Disease prediction is one of the main models for the development of medicine in the 21st century, and it has become an important component of the clinical research methodology system to construct disease risk intelligent prediction models. Currently, real-world electronic medical records (EMRs) have become an important data foundation for disease risk intelligent prediction research. However, not all collections of real-world EMRs are suitable for building disease risk intelligent prediction research, so there is an urgent need for a set of indicators to guide the evaluation of whether a certain EMR dataset is suitable for building disease risk prediction models.

The purpose of this questionnaire is to establish an evaluation index system for the applicability of EMR data to construct disease risk intelligent prediction models. Given your profound expertise in this field, we sincerely invite you to participate as an expert in this expert consultation. Adheres to scientific research principles, your information will be kept confidential.

The expert consultation questionnaire is divided into four parts. The first part is a survey of basic information about the expert. The second part is a survey on the expert's familiarity with the content and judgment criteria. The third part is an expert consultation on the initial evaluation index system for the applicability of electronic medical record data in disease risk intelligent prediction research (referred to as the initial evaluation index system). The initial evaluation index system includes 4 first-level indicators, 11 second-level indicators, and 33 third-level indicators. The fourth part is an expert consultation on the calculation methods for the third-level indicators in the initial evaluation index system.

We would like to request your opinion on the following matters:

1. Regarding the initial evaluation index system shown in Table S4-S6, could you evaluate: (1) whether the evaluation index system is comprehensive and whether any necessary indicators are missing; (2) whether the naming and definitions of indicators are accurate; and (3) the relative importance of indicators.

2. Regarding the calculation method for the third-level indicators shown in Table S7, could you evaluate whether the calculation method is feasible and acceptable, and if any modifications are needed, please provide corresponding suggestions.

To ensure the progress of the study, we kindly request that you email the completed questionnaire form to [804964646@qq.com](mailto:804964646@qq.com) within one week. We sincerely look forward to and appreciate your opinions and suggestions.

Once again, thank you for your support of this research!

**Part I: Survey of Experts’ Basic Information Form**

**Instructions**: This questionnaire is for research purposes only, and we guarantee that we will keep all completed information confidential. Please fill in the corresponding cells according to your own situation. If there is additional information to be added, please make a separate note. Thank you!

**Table S1. Experts’ Basic Information**

| Name |  | Gender |  | Age |  |
| --- | --- | --- | --- | --- | --- |
| Affiliation |  | | | | |
| Job Title |  | Job Position |  | Education Level |  |
| Field of Expertise |  | | Years of Professional Work Experience |  | |
| Contact Information |  | | | | |
| Mailing Address |  | | | | |

**Part II: Survey of Experts’ Familiarity with Consultation Content and Judgment Basis Form**

**Instructions:**

1. Table S2 is mainly used to understand your familiarity with the consulting content. Please mark “√” in the corresponding cell of familiarity.

2. Table S3 is mainly used to understand the influence degree of relevant judgment criteria on your opinions and suggestions for this consultation. The judgment criteria for this research consultation are divided into theoretical analysis, practical experience, domestic and international materials, and personal subjective judgment, and their influence degree is divided into three levels: high, medium, and low. Please mark “√” in the corresponding cell of the influence degree of the 4 judgment criteria on your opinions and suggestions for this consultation.

**Table S2. Experts’ Familiarity with Consultation Content**

| **Familiarity** | **Very Familiar** | **Relatively Familiar** | **General** | **Relatively Unfamiliar** | **Very Unfamiliar** |
| --- | --- | --- | --- | --- | --- |
| **Self-Assessment** |  |  |  |  |  |

**Table S3. Judgement Basis and Influence Degree**

| **Basis** | **Theoretical Analysis** | | | **Practical Experience** | | | **Domestic and International Materials** | | | **Subjective Judgment** | | |
| --- | --- | --- | --- | --- | --- | --- | --- | --- | --- | --- | --- | --- |
| **Degree** | **High** | **Medium** | **Low** | **High** | **Medium** | **Low** | **High** | **Medium** | **Low** | **High** | **Medium** | **Low** |
| **Result** |  |  |  |  |  |  |  |  |  |  |  |  |

**Part III: Expert Consultation Form for the Indicator System**

**Instructions:**

1. Tables S4-S6 are the consultation forms for the first-level, second-level, and third-level indicators, respectively. Please evaluate the importance level of each indicator in the context of disease risk intelligent prediction research, based on your own judgement criteria, and mark “√” in the corresponding column of the “Importance Evaluation” section.

2. If you think that the indicator names or descriptions are not reasonable, please mark “√” in the "Deletion" column and provide your reasons in the "Suggestions for Modification" column. Alternatively, you can suggest modified indicator names or descriptions in the "Suggestions for Modification" column.

3. If you think that an important indicator has not been included, please use the blank rows below "Suggested Additional Indicators" in Tables S4-S6 to provide the necessary information. You can add rows as needed and include the indicator name, description, and importance evaluation.

**Table S4. Expert Consultation Form on First-Level Indicators**

| **First-Level Indicators** | **Description** | **Importance Evaluation** | | | | | **Deletion** | **Suggestions for Modification** |
| --- | --- | --- | --- | --- | --- | --- | --- | --- |
|  |  | **Very Important** | **Relatively Important** | **General** | **Relatively Unimportant** | **Very Unimportant** |  |  |
| **Operability** | The degree or proportion to which each part of the EMR dataset in use can satisfy the selection, transformation, compression, transplantation, integration, or other processing operations required by the predictive model. |  |  |  |  |  |  |  |
| **Completeness** | The level of completeness of the data in use in terms of data elements, data element values, and other aspects required by the predictive model. |  |  |  |  |  |  |  |
| **Correctness** | A metric for the compliance, accuracy, and effectiveness of the data elements required by the predictive model. |  |  |  |  |  |  |  |
| **Timeliness** | The temporal characteristics of the EMR data in use meet the expected requirements of the predictive model. |  |  |  |  |  |  |  |
| **Suggested Additional Indicators** | | | | | | | | |
|  |  |  |  |  |  |  |  |  |

**Table S5. Expert Consultation Form on Second-Level Indicators**

| **First-Level Indicators** | **Second- Level Indicators** | **Description** | **Importance Evaluation** | | | | | **Deletion** | **Suggestions for Modification** |
| --- | --- | --- | --- | --- | --- | --- | --- | --- | --- |
|  |  |  | **Very Important** | **Relatively Important** | **General** | **Relatively Unimportant** | **Very Unimportant** |  |  |
| **Operability** | **Integrability** | Each part of the EMR dataset in use can be horizontally merged, vertically merged, or used for transfer learning. |  |  |  |  |  |  |  |
|  | **Portability** | The characteristic of the dataset being able to be transferred from one hardware or software environment to another. |  |  |  |  |  |  |  |
|  | **Selectivity** | The convenience of the data in use for predictive algorithms to select parameters or features that meet the requirements of the predictive model. |  |  |  |  |  |  |  |
|  | **Suggested Additional Indicators** | | | | | | | | |
|  |  |  |  |  |  |  |  |  |  |
| **Completeness** | **Integrity of data elements** | The level of completeness of the data elements and their corresponding values for the input and output parameters required by the predictive model. |  |  |  |  |  |  |  |
|  | **Integrity of temporal information** | The level of completeness of time information in the data element values corresponding to the parameters required by the predictive model. |  |  |  |  |  |  |  |
|  | **Integrity of data state** | The level of completeness of the status of the parameters required by the predictive model. |  |  |  |  |  |  |  |
|  | **Data balance** | The necessary level of the expected distribution of features in the EMR data required by the predictive algorithm. |  |  |  |  |  |  |  |
|  | **Suggested Additional Indicators** | | | | | | | | |
|  |  |  |  |  |  |  |  |  |  |
| **Correctness** | **Data accuracy** | The degree to which the required data elements for the predictive model reflect the actual situation. |  |  |  |  |  |  |  |
|  | **Data consistency** | The degree of consistency or absence of conflicts in the data required by the predictive model across different parts of the dataset. |  |  |  |  |  |  |  |
|  | **Data compliance** | The level to which the data required by the predictive model complies with the technical standards or specifications claimed by the data provider. |  |  |  |  |  |  |  |
|  | **Suggested Additional Indicators** | | | | | | | | |
|  |  |  |  |  |  |  |  |  |  |
| **Timeliness** | **Data timeliness** | The EMR data in use is able to reflect the actual and latest state. |  |  |  |  |  |  |  |
|  | **Suggested Additional Indicators** | | | | | | | | |
|  |  |  |  |  |  |  |  |  |  |

**Table S6. Expert Consultation Form on Third-Level Indicators**

| **Second- Level Indicators** | **Third-Level Indicators** | **Description** | **Importance Evaluation** | | | | | **Deletion** | **Suggestions for Modification** |
| --- | --- | --- | --- | --- | --- | --- | --- | --- | --- |
|  |  |  | **Very Important** | **Relatively Important** | **General** | **Relatively Unimportant** | **Very Unimportant** |  |  |
| **Integrability** | **Ratio of mapping the primary key** | The data records between each part of the EMR dataset in use can be mapped one-to-one through a primary key. |  |  |  |  |  |  |  |
|  | **Ratio of mapping data elements** | The proportion of data elements used in model prediction that can be mapped between databases. |  |  |  |  |  |  |  |
|  | **Ratio of interconvertible data elements** | The proportion of the EMR dataset in use that can be transformed according to the requirements of the predictive model. |  |  |  |  |  |  |  |
|  | **Suggested Additional Indicators** | | | | | | | | |
|  |  |  |  |  |  |  |  |  |  |
| **Portability** | **Reliable data migration** | The data records of each part of the EMR dataset in use can be accurately replicated. |  |  |  |  |  |  |  |
|  | **Suggested Additional Indicators** | | | | | | | | |
|  |  |  |  |  |  |  |  |  |  |
| **Selectivity** | **Sufficient data elements as inputs for feature engineering** | The convenience of the data in use for predictive algorithms to select or combine input parameters. |  |  |  |  |  |  |  |
|  | **Sufficient data elements as outputs for feature engineering** | The convenience of the data in use for predictive algorithms to select or combine output parameters. |  |  |  |  |  |  |  |
|  | **Measurement selectivity in data inputs for feature engineering** | The degree to which the data in use can be selected for data records based on the values of input parameter features by predictive algorithms. |  |  |  |  |  |  |  |
|  | **Measurement selectivity in data outputs for feature engineering** | The degree to which the data in use can be selected for data records based on the values of output parameter features by predictive algorithms. |  |  |  |  |  |  |  |
|  | **Suggested Additional Indicators** | | | | | | | | |
|  |  |  |  |  |  |  |  |  |  |
| **Integrity of data elements** | **Integrity of data elements as inputs in predictive modeling** | Completeness of the set of data elements corresponding to the input parameters of the prediction model. |  |  |  |  |  |  |  |
|  | **Integrity of data elements as outputs in predictive modeling** | The completeness of the set of data elements corresponding to the output parameters of the prediction model. |  |  |  |  |  |  |  |
|  | **Integrity of data values as inputs in predictive modeling** | The extent to which the set of data elements needed to predict the input parameters of the model take values that are not null or free of missing. |  |  |  |  |  |  |  |
|  | **Integrity of data values as outputs in predictive modeling** | Predict the completeness of the set of data elements needed to take the values of the output parameters. |  |  |  |  |  |  |  |
|  | **Suggested Additional Indicators** | | | | | | | | |
|  |  |  |  |  |  |  |  |  |  |
| **Integrity of temporal information** | **Integrity of timestamps as creating data** | The degree of completeness of the generation time of the data elements corresponding to the parameters required by the prediction model. |  |  |  |  |  |  |  |
|  | **Integrity of timestamps as creating values with data** | The degree of completeness of the generation time of the data element fetching values corresponding to the parameters required by the prediction model. |  |  |  |  |  |  |  |
|  | **Suggested Additional Indicators** | | | | | | | | |
|  |  |  |  |  |  |  |  |  |  |
| **Integrity of data state** | **Integrity of data state in available** | The parameters needed for the predictive model have complete data state information. |  |  |  |  |  |  |  |
|  | **Suggested Additional Indicators** | | | | | | | | |
|  |  |  |  |  |  |  |  |  |  |
| **Data balance** | **Adequate data** | The number of available and unique electronic medical record data records expected to be available is capable of reaching the necessary level required by the prediction algorithm. |  |  |  |  |  |  |  |
|  | **Balance of input data** | The extent to which the diversity of predictor variables included in the data matches the actual clinical scenario required by the prediction task. |  |  |  |  |  |  |  |
|  | **Balance of output data** | The diversity of outcome variables included in the data is consistent with the actual clinical scenario required by the prediction task. |  |  |  |  |  |  |  |
|  | **Suggested Additional Indicators** | | | | | | | | |
|  |  |  |  |  |  |  |  |  |  |
| **Data accuracy** | **Accurate data formats** | The data format required by the predictive model, such as csv, meets the requirements for data processing and analysis. |  |  |  |  |  |  |  |
|  | **Accurate data types** | The data type of the data element values required by the predictive model matches the corresponding data element. |  |  |  |  |  |  |  |
|  | **Right level of granularity** | The level to which the precision of the data elements and their values required by the predictive model meets the level claimed by the data provider. |  |  |  |  |  |  |  |
|  | **Accurate measurement of data** | The degree to which the data element values required by the predictive model can reflect the actual situation. |  |  |  |  |  |  |  |
|  | **Unambiguity of data elements** | The degree of uniqueness of the data elements required by the predictive model within the dataset, meaning that there are no data elements with the same name but different meanings. |  |  |  |  |  |  |  |
|  | **Unambiguity of measurement of data** | The unambiguity level of the data element values required by the predictive model in the dataset, meaning that there are no data element value with the same name but a different meaning. |  |  |  |  |  |  |  |
|  | **Suggested Additional Indicators** | | | | | | | | |
|  |  |  |  |  |  |  |  |  |  |
| **Data consistency** | **Consistent measurement of data** | The proportion of data element values, such as default values or recorded values, remain consistent to the same fact. |  |  |  |  |  |  |  |
|  | **Consistent metric calculations** | All values under the same data element required by the predictive model have the same calculation method. |  |  |  |  |  |  |  |
|  | **Consistent metric units** | The values of data elements required by the predictive model have consistent units. |  |  |  |  |  |  |  |
|  | **Suggested Additional Indicators** | | | | | | | | |
|  |  |  |  |  |  |  |  |  |  |
| **Data compliance** | **Data elements for compliance** | The degree to which the naming, definition, and other descriptions of the data elements required by the predictive model are consistent with the technical standards or specifications claimed by the data provider. |  |  |  |  |  |  |  |
|  | **Data measurement for compliance** | The degree to which the data element values required by the predictive model comply with the technical standards or specifications claimed by the data provider. |  |  |  |  |  |  |  |
|  | **Standard timestamps for compliance** | The level of clinical reasonableness or interpretability of temporal information in the data related to dynamic temporal processes involved in clinical diagnosis and treatment, in terms of clinical timeliness. |  |  |  |  |  |  |  |
|  | **Standard time logs for compliance** | The level of clinical reasonableness or meaningfulness of the duration of data records related to dynamic temporal processes involved in clinical diagnosis and treatment. |  |  |  |  |  |  |  |
|  | **Suggested Additional Indicators** | | | | | | | | |
|  |  |  |  |  |  |  |  |  |  |
| **Data timeliness** | **Timeliness on recording data** | The degree to which the EMRs in use are recorded in a timely manner. |  |  |  |  |  |  |  |
|  | **Frequency on recording data** | The level of completeness of the values of data elements corresponding to each time point of the parameters requiring continuous time records required by the predictive model. |  |  |  |  |  |  |  |
|  | **Suggested Additional Indicators** | | | | | | | | |
|  |  |  |  |  |  |  |  |  |  |

**Part IV: Expert Consultation Form on Calculation Methods for Third-Level Indicators**

**Instructions:** Table S7 presents the definitions and quantitative calculation formulas for the initial third-level indicators. Please evaluate the acceptability of the calculation formulas for the initial third-level indicators. If you think that the calculation formula is acceptable, please mark “√” in the “Acceptable” column. Otherwise, mark “√” in the “Needs Modification” column and provide your modification suggestions in the “Suggestions for Modification” column.

**Table S7. Expert Consultation Form on the Acceptability of Quantitative Calculation Formulas for Third-Level Indicators**

| **Third-Level Indicators** | **Description** | **Calculation Formula** | **Acceptable** | **Needs Modification** | **Suggestions for Modification** |
| --- | --- | --- | --- | --- | --- |
| **Ratio of mapping the primary key** | The data records between each part of the EMR dataset in use can be mapped one-to-one through a primary key. | Number of mappable primary key records / Total number of primary key records expected to be mapped |  |  |  |
| **Ratio of mapping data elements** | The proportion of data elements used in model prediction that can be mapped between databases. | Number of mappable data elements / Total number of data elements expected to be mapped (Note: these counts refer to the number of distinct data elements) |  |  |  |
| **Ratio of interconvertible data elements** | The proportion of the EMR dataset in use that can be transformed according to the requirements of the predictive model. | Number of data elements that can be mutually converted / Total number of data elements expected to be converted |  |  |  |
| **Reliable data migration** | The data records of each part of the EMR dataset in use can be accurately replicated. | Number of replicable records with accurate results / Total number of records |  |  |  |
| **Sufficient data elements as inputs for feature engineering** | The convenience of the data in use for predictive algorithms to select or combine input parameters. | Number of selectable input data elements / Total number of input data elements |  |  |  |
| **Sufficient data elements as outputs for feature engineering** | The convenience of the data in use for predictive algorithms to select or combine output parameters. | Number of selectable output data elements / Total number of output data elements |  |  |  |
| **Measurement selectivity in data inputs for feature engineering** | The degree to which the data in use can be selected for data records based on the values of input parameter features by predictive algorithms. | Number of selectable input parameter feature measurements / Total number of input parameter feature measurements |  |  |  |
| **Measurement selectivity in data outputs for feature engineering** | The extent to which the data put into use can be used by the prediction algorithm for data record selection based on the values taken for the output class parameter features | Number of selectable output parameter feature measurements / Total number of output parameter feature measurements |  |  |  |
| **Integrity of data elements as inputs in predictive modeling** | Completeness of the set of data elements corresponding to the input parameters of the prediction model | Number of input parameter data elements / Expected total input parameter data elements |  |  |  |
| **Integrity of data elements as outputs in predictive modeling** | The completeness of the set of data elements corresponding to the output parameters of the prediction model | Number of output parameter data elements / Expected total output parameter data elements |  |  |  |
| **Integrity of data values as inputs in predictive modeling** | The extent to which the set of data elements needed to predict the input parameters of the model take values that are not empty or free of missing | Number of data records without missing values in input feature set / Total number of data records with values in input feature set |  |  |  |
| **Integrity of data values as outputs in predictive modeling** | Predict the completeness of the set of data elements needed to take the values of the output parameters | Number of data records without missing values in output feature set / Total number of data records with values in output feature set |  |  |  |
| **Integrity of timestamps as creating data** | The degree of completeness of the generation time of the data elements corresponding to the parameters required by the prediction model | Number of data elements with time generation or update information / Total number of data elements used in predictive model |  |  |  |
| **Integrity of timestamps as creating values with data** | The degree of completeness of the generation time of the data element fetching values corresponding to the parameters required by the prediction model | Number of data values with time generation or update information / Total number of data values used in predictive model |  |  |  |
| **Integrity of data state in available** | The parameters needed for the predictive model have complete data state information | A value of 1 is assigned if the data record is available, and 0 otherwise |  |  |  |
| **Adequate data** | The number of available and unique electronic medical record data records expected to be available is capable of reaching the necessary level required by the prediction algorithm | A value of 1 is assigned if the number of data records is greater than or equal to the number required by the algorithm, and 0 otherwise |  |  |  |
| **Balance of input data** | The extent to which the diversity of predictor variables included in the data matches the actual clinical scenario required by the prediction task | Number of independent variables required for prediction covered by electronic medical records (EMRs) in use / Total number of independent variables required by the predictive model |  |  |  |
| **Balance of output data** | The diversity of outcome variables included in the data is consistent with the actual clinical scenario required by the prediction task | Number of dependent variables required for prediction covered by electronic medical records (EMRs) in use / Total number of dependent variables required by the predictive model |  |  |  |
| **Accurate data formats** | The data format required by the predictive model, such as csv, meets the requirements for data processing and analysis | A value of 1 is assigned if the data format meets the requirements, and 0 otherwise |  |  |  |
| **Accurate data types** | The data types of the data element values required by the prediction model correspond to the data element counterparts | Number of data element values with correct data types / Total number of data element values required by predictive model |  |  |  |
| **Right level of granularity** | The extent to which the precision of the data elements and data element values required by the prediction model meets the level claimed by the data provider | Number of data elements with accurate expression / Total number of data elements |  |  |  |
| **Accurate measurement of data** | The metadata needed for the predictive model takes values that reflect the actual situation | Number of data element measurements required for a logical prediction model / Total number of data element measurements required for prediction |  |  |  |
| **Unambiguity of data elements** | The degree of uniqueness within the dataset of the data elements required by the prediction model, i.e., no data elements with the same name but different actual meanings | 1 – (Number of ambiguous data elements after deduplication / Total number of deduplicated data elements) |  |  |  |
| **Unambiguity of measurement of data** | The degree of ambiguity within the dataset for the values of the data elements required by the prediction model, i.e., there are no data elements with the same name but different actual meanings | 1 – (Number of ambiguous data element measurements / Total number of data element measurements) |  |  |  |
| **Consistent measurement of data** | Percentage of data elements with the same description of the same fact and consistent default or record values | Number of data elements in data records that conform to rules / Total number of data elements |  |  |  |
| **Consistent metric calculations** | All values under the same data element required by the prediction model are calculated in the same way | A value of 1 is assigned if all values of the same data element have the same calculation method, and 0 otherwise |  |  |  |
| **Consistent metric units** | The prediction requires the same unit of value under the data element | A value of 1 is assigned if the measurement units are consistent, and 0 otherwise |  |  |  |
| **Data elements for compliance** | The extent to which the descriptions of data element naming, definitions, etc. required by the predictive model conform to the technical standards or specifications claimed by the data provider | Number of compliant data elements / Total number of data elements required for predictive model |  |  |  |
| **Data measurement for compliance** | The extent to which the data elements required by the predictive model are taken to conform to the technical standards or specifications claimed by the data provider | Number of compliant data element measurements / Total number of data element measurements |  |  |  |
| **Standard timestamps for compliance** | The degree to which data timing information involving dynamic timing processes in clinical diagnosis and treatment is reasonable or interpretable in terms of clinical timeliness | Number of data records that comply the time logic / Total number of data records with temporal information |  |  |  |
| **Standard time logs for compliance** | The extent to which it is clinically reasonable or meaningful to record the duration of data involving a dynamic time-series process in clinical diagnosis and treatment | Number of compliant data elements related to dynamic temporal processes / Total number of data elements required for predictive model |  |  |  |
| **Timeliness on recording data** | The extent to which data from electronic medical records put into use are recorded in a timely manner | Number of data records that meet time requirements / Total number of data records |  |  |  |
| **Frequency on recording data** | The degree of completeness of the data elements taken for each time node corresponding to the parameters of the continuous time records required by the prediction model | Number of continuous time record parameters with values at different time points / Total number of values for continuous parameter |  |  |  |
