# Supplementary material for: Development of a quantitative index system for evaluating the quality of electronic medical records in disease risk intelligent prediction
Source: BMC Med Inform Decis Mak. 2024 Jun 24;24:178. doi: 10.1186/s12911-024-02533-z (PMC11194906; doi:10.1186/s12911-024-02533-z)
Supplement: Supplementary file 2 — Supplementary Material 2. [file 12911_2024_2533_MOESM2_ESM.docx]

**Additional file 2**

The Quantitative Three-Level Index System for Evaluating the Quality of Electronic Medical Records (EMRs) in Predictive Modeling

| **Code** | **First-Level Indicator** | **Second-Level Indicator** | **Third-Level Indicator** | **Definition** | **Range of Values** | **Weight** |
| --- | --- | --- | --- | --- | --- | --- |
| 1 | Operability |  |  | The degree or proportion to which each part of the EMR dataset in use can satisfy the selection, transformation, compression, transplantation, integration, or other processing operations required by the predictive model. | [0, 1] | 0.251 |
| 1.1 |  | Integrability |  | Each part of the EMR dataset in use can be horizontally merged, vertically merged, or used for transfer learning. | [0, 1] | 0.091 |
| 1.1.1 |  |  | Ratio of mapping the primary key | The data records between each part of the EMR dataset in use can be mapped one-to-one through a primary key. | [0, 1] | 0.032 |
| 1.1.2 |  |  | Ratio of mapping data elements | The proportion of data elements used in model prediction that can be mapped between databases. | [0, 1] | 0.030 |
| 1.1.3 |  |  | Ratio of interconvertible data elements | The proportion of the EMR dataset in use that can be transformed according to the requirements of the predictive model. | [0, 1] | 0.029 |
| 1.2 |  | Portability |  | The characteristic of the dataset being able to be transferred from one hardware or software environment to another. | 0 or 1 | 0.082 |
| 1.2.1 |  |  | Reliable data migration | The data records of each part of the EMR dataset in use can be accurately replicated. | 0 or 1 | 0.082 |
| 1.3 |  | Selectivity |  | The convenience of the data in use for predictive algorithms to select parameters or features that meet the requirements of the predictive model. | [0, 1] | 0.078 |
| 1.3.1 |  |  | Sufficient data elements as inputs for feature engineering | The convenience of the data in use for predictive algorithms to select or combine input parameters. | [0, 1] | 0.020 |
| 1.3.2 |  |  | Sufficient data elements as outputs for feature engineering | The convenience of the data in use for predictive algorithms to select or combine output parameters. | [0, 1] | 0.019 |
| 1.3.3 |  |  | Measurement selectivity in data inputs for feature engineering | The degree to which the data in use can be selected for data records based on the values of input parameter features by predictive algorithms. | [0, 1] | 0.020 |
| 1.3.4 |  |  | Measurement selectivity in data outputs for feature engineering | The degree to which the data in use can be selected for data records based on the values of output parameter features by predictive algorithms. | [0, 1] | 0.019 |
| 2 | Completeness |  |  | The level of completeness of the data in use in terms of data elements, data element values, and other aspects required by the predictive model. | [0, 1] | 0.254 |
| 2.1 |  | Integrity of data elements |  | The level of completeness of the data elements and their corresponding values for the input and output parameters required by the predictive model. | [0, 1] | 0.066 |
| 2.1.1 |  |  | Integrity of data elements as inputs in predictive modeling | The level of completeness of the set of data elements corresponding to the input parameters of the predictive model. | [0, 1] | 0.017 |
| 2.1.2 |  |  | Integrity of data elements as outputs in predictive modeling | The level of completeness of the set of data elements corresponding to the output parameters of the predictive model. | [0, 1] | 0.016 |
| 2.1.3 |  |  | Integrity of data values as inputs in predictive modeling | The degree to which the values of the data elements required for the input parameters of the predictive model are not null or missing. | [0, 1] | 0.017 |
| 2.1.4 |  |  | Integrity of data values as outputs in predictive modeling | The degree to which the values of the data elements required for the output parameters of the predictive model are complete. | [0, 1] | 0.016 |
| 2.2 |  | Integrity of temporal information |  | The level of completeness of time information in the data element values corresponding to the parameters required by the predictive model. | [0, 1] | 0.062 |
| 2.2.1 |  |  | Integrity of timestamps as creating data | The level of completeness of the generation time of the data elements corresponding to the parameters required by the predictive model. | [0, 1] | 0.032 |
| 2.2.2 |  |  | Integrity of timestamps as creating values with data | The level of completeness of the generation time information in the data element values corresponding to the parameters required by the predictive model. | [0, 1] | 0.031 |
| 2.3 |  | Integrity of data state |  | The level of completeness of the status of the parameters required by the predictive model. | 0 or 1 | 0.063 |
| 2.3.1 |  |  | Integrity of data state in available | The level of completeness of the status information of the parameters required by the predictive model. | 0 or 1 | 0.063 |
| 2.4 |  | Data balance |  | The necessary level of the expected distribution of features in the EMR data required by the predictive algorithm. | [0, 1] | 0.063 |
| 2.4.1 |  |  | Adequate data | The necessary level of the expected number of unique EMR data records in available required by the predictive algorithm. | 0 or 1 | 0.020 |
| 2.4.2 |  |  | Balance of input data | The degree to which the diversity of independent variables contained in the data conforms to the clinical reality required by the predictive task. | [0, 1] | 0.021 |
| 2.4.3 |  |  | Balance of output data | The degree to which the diversity of dependent variables contained in the data conforms to the clinical reality required by the predictive task. | 0 or 1 | 0.022 |
| 3 | Correctness |  |  | A metric for the compliance, accuracy, and effectiveness of the data elements required by the predictive model. | [0, 1] | 0.264 |
| 3.1 |  | Data accuracy |  | The degree to which the required data elements for the predictive model reflect the actual situation. | [0, 1] | 0.087 |
| 3.1.1 |  |  | Accurate data formats | The format of dataset required by the predictive model, such as CSV, meets the requirements of data processing and analysis. | 0 or 1 | 0.013 |
| 3.1.2 |  |  | Accurate data types | The data type of the data element values required by the predictive model matches the corresponding data element. | [0, 1] | 0.015 |
| 3.1.3 |  |  | Right level of granularity | The level to which the precision of the data elements and their values required by the predictive model meets the level claimed by the data provider. | [0, 1] | 0.015 |
| 3.1.4 |  |  | Accurate measurement of data | The degree to which the data element values required by the predictive model can reflect the actual situation. | [0, 1] | 0.015 |
| 3.1.5 |  |  | Unambiguity of data elements | The degree of uniqueness of the data elements required by the predictive model within the dataset, meaning that there are no data elements with the same name but different meanings. | [0, 1] | 0.015 |
| 3.1.6 |  |  | Unambiguity of measurement of data | The unambiguity level of the data element values required by the predictive model in the dataset, meaning that there are no data element value with the same name but a different meaning. | [0, 1] | 0.015 |
| 3.2 |  | Data consistency |  | The degree of consistency or absence of conflicts in the data required by the predictive model across different parts of the dataset. | [0, 1] | 0.089 |
| 3.2.1 |  |  | Consistent measurement of data | The proportion of data element values, such as default values or recorded values, remain consistent to the same fact. | [0, 1] | 0.031 |
| 3.2.2 |  |  | Consistent metric calculations | All values under the same data element required by the predictive model have the same calculation method. | 0 or 1 | 0.030 |
| 3.2.3 |  |  | Consistent metric units | The values of data elements required by the predictive model have consistent units. | 0 or 1 | 0.028 |
| 3.3 |  | Data compliance |  | The level to which the data required by the predictive model complies with the technical standards or specifications claimed by the data provider. | [0, 1] | 0.089 |
| 3.3.1 |  |  | Data elements for compliance | The degree to which the naming, definition, and other descriptions of the data elements required by the predictive model are consistent with the technical standards or specifications claimed by the data provider. | [0, 1] | 0.023 |
| 3.3.2 |  |  | Data measurement for compliance | The degree to which the data element values required by the predictive model comply with the technical standards or specifications claimed by the data provider. | [0, 1] | 0.022 |
| 3.3.3 |  |  | Standard timestamps for compliance | The level of clinical reasonableness or interpretability of temporal information in the data related to dynamic temporal processes involved in clinical diagnosis and treatment, in terms of clinical timeliness. | [0, 1] | 0.022 |
| 3.3.4 |  |  | Standard time logs for compliance | The level of clinical reasonableness or meaningfulness of the duration of data records related to dynamic temporal processes involved in clinical diagnosis and treatment. | [0, 1] | 0.023 |
| 4 | Timeliness |  |  | The temporal characteristics of the EMR data in use meet the expected requirements of the predictive model. | [0, 1] | 0.231 |
| 4.1 |  | Data timeliness |  | The EMR data in use is able to reflect the actual and latest state. | [0, 1] | 0.231 |
| 4.1.1 |  |  | Timeliness on recording data | The degree to which the EMRs in use are recorded in a timely manner. | [0, 1] | 0.113 |
| 4.1.2 |  |  | Frequency on recording data | The level of completeness of the values of data elements corresponding to each time point of the parameters requiring continuous time records required by the predictive model. | [0, 1] | 0.118 |

Calculation Formulas for Assigning Weights to Third-Level Indicators

| **Code** | **Third-Level Indicator** | **Formula** |
| --- | --- | --- |
| 1.1.1 | Ratio of mapping the primary key | Number of mappable primary key records / Total number of primary key records expected to be mapped |
| 1.1.2 | Ratio of mapping data elements | Number of mappable data elements / Total number of data elements expected to be mapped (Note: these counts refer to the number of distinct data elements) |
| 1.1.3 | Ratio of interconvertible data elements | Number of data elements that can be mutually converted / Total number of data elements expected to be converted |
| 1.2.1 | Reliable data migration | Number of replicable records with accurate results / Total number of records |
| 1.3.1 | Sufficient data elements as inputs for feature engineering | Number of selectable input data elements / Total number of input data elements |
| 1.3.2 | Sufficient data elements as outputs for feature engineering | Number of selectable output data elements / Total number of output data elements |
| 1.3.3 | Measurement selectivity in data inputs for feature engineering | Number of selectable input parameter feature measurements / Total number of input parameter feature measurements |
| 1.3.4 | Measurement selectivity in data outputs for feature engineering | Number of selectable output parameter feature measurements / Total number of output parameter feature measurements |
| 2.1.1 | Integrity of data elements as inputs in  predictive modeling | Number of input parameter data elements / Expected total input parameter data elements |
| 2.1.2 | Integrity of data elements as outputs in  predictive modeling | Number of output parameter data elements / Expected total output parameter data elements |
| 2.1.3 | Integrity of data values as inputs in  predictive modeling | Number of data records without missing values in input feature set / Total number of data records with values in input feature set |
| 2.1.4 | Integrity of data values as outputs in  predictive modeling | Number of data records without missing values in output feature set / Total number of data records with values in output feature set |
| 2.2.1 | Integrity of timestamps as creating data | Number of data elements with time generation or update information / Total number of data elements used in predictive model |
| 2.2.2 | Integrity of timestamps as creating values with data | Number of data values with time generation or update information / Total number of data values used in predictive model |
| 2.3.1 | Integrity of data state in available | A value of 1 is assigned if the data record is available, and 0 otherwise |
| 2.4.1 | Adequate data | A value of 1 is assigned if the number of data records is greater than or equal to the number required by the algorithm, and 0 otherwise |
| 2.4.2 | Balance of input data | Number of independent variables required for prediction covered by electronic medical records (EMRs) in use / Total number of independent variables required by the predictive model |
| 2.4.3 | Balance of output data | Number of dependent variables required for prediction covered by electronic medical records (EMRs) in use / Total number of dependent variables required by the predictive model |
| 3.1.1 | Accurate data formats | A value of 1 is assigned if the data format meets the requirements, and 0 otherwise |
| 3.1.2 | Accurate data types | Number of data element values with correct data types / Total number of data element values required by predictive model |
| 3.1.3 | Right level of granularity | Number of data elements with accurate expression / Total number of data elements |
| 3.1.4 | Accurate measurement of data | Number of data element measurements required for a logical prediction model / Total number of data element measurements required for prediction |
| 3.1.5 | Unambiguity of data elements | 1 – (Number of ambiguous data elements after deduplication / Total number of deduplicated data elements) |
| 3.1.6 | Unambiguity of measurement of data | 1 – (Number of ambiguous data element measurements / Total number of data element measurements) |
| 3.2.1 | Consistent measurement of data | Number of data elements in data records that conform to rules / Total number of data elements |
| 3.2.2 | Consistent metric calculations | A value of 1 is assigned if all values of the same data element have the same calculation method, and 0 otherwise |
| 3.2.3 | Consistent metric units | A value of 1 is assigned if the measurement units are consistent, and 0 otherwise |
| 3.3.1 | Data elements for compliance | Number of compliant data elements / Total number of data elements required for predictive model |
| 3.3.2 | Data measurement for compliance | Number of compliant data element measurements / Total number of data element measurements |
| 3.3.3 | Standard timestamps for compliance | Number of data records that comply the time logic / Total number of data records with temporal information |
| 3.3.4 | Standard time logs for compliance | Number of compliant data elements related to dynamic temporal processes / Total number of data elements required for predictive model |
| 4.1.1 | Timeliness on recording data | Number of data records that meet time requirements / Total number of data records |
| 4.1.2 | Frequency on recording data | Number of continuous time record parameters with values at different time points / Total number of values for continuous parameter |
