# Supplementary material for: Development of a quantitative index system for evaluating the quality of electronic medical records in disease risk intelligent prediction
Source: BMC Med Inform Decis Mak. 2024 Jun 24;24:178. doi: 10.1186/s12911-024-02533-z (PMC11194906; doi:10.1186/s12911-024-02533-z)
Supplement: Supplementary file 3 — Supplementary Material 3. [file 12911_2024_2533_MOESM3_ESM.docx]

**Additional file 3**

**Table S8.** The characteristics of the participated experts

| **Experts' information** | | **Count (n)** | **Percentage (%)** |
| --- | --- | --- | --- |
| **Gender** | Male | 13 | 81.25 |
|  | Female | 3 | 18.75 |
| **Age (in years)** | <40 | 6 | 37.5 |
|  | 40-50 | 9 | 56.25 |
|  | >= 50 | 1 | 6.25 |
| **Working experience (in years)** | <10 | 3 | 18.75 |
|  | 10-20 | 10 | 62.5 |
|  | >20 | 3 | 18.75 |
| **Education** | Bachelor's degree | 1 | 6.25 |
|  | Master's degree | 3 | 18.75 |
|  | Ph.D. | 12 | 75 |
| **Professional title** | Assistant | 5 | 31.25 |
|  | Associate senior or above | 11 | 68.75 |
| **Area of expertise** | Medical Informatics | 6 | 37.5 |
|  | Clinical Research Methods | 2 | 12.5 |
|  | Clinical Comprehensive Evaluation | 1 | 6.25 |
|  | Regional Medical Informatization | 3 | 18.75 |
|  | Medical AI | 4 | 25 |

**Table S9.** Statistics on the basis of expert judgment

|  | **High** | **Medium** | **Low** |
| --- | --- | --- | --- |
| **Theoretical Analysis** | 6 | 10 | 0 |
| **Practical Experience** | 12 | 4 | 0 |
| **Domestic and International Materials** | 7 | 7 | 2 |
| **Subjective Judgment** | 3 | 11 | 2 |

**Table S10.** Basic information of the five datasets

| **Dataset** | **Population** | **No. of Patients** | **No. of Sepsis** | **No. of Non-Sepsis** |
| --- | --- | --- | --- | --- |
| **Elderly** | Elderly patients | 10,048 | 3,728 | 6,320 |
| **LLOS** | Long length of stay patients | 1,375 | 578 | 797 |
| **Stroke** | Ischemic stroke patients | 1,024 | 250 | 774 |
| **ARF** | Acute renal failure patients | 634 | 350 | 284 |
| **CIR** | Cirrhotic patients | 430 | 137 | 293 |

**Table S11.** Scoring results of divergent indicators

| **Dataset** | **2.1.3^1^** | **2.2.2^2^** | **2.4.1^3^** | **3.1.4^4^** | **4.1.1^5^** | **4.1.2^6^** |
| --- | --- | --- | --- | --- | --- | --- |
| **Elderly** | 0.923 | 0.923 | 0 | 0.691 | 0.904 | 0.614 |
| **LLOS** | 0.423 | 0.423 | **1** | 0.983 | **0.949** | **0.996** |
| **Stroke** | 0.913 | 0.913 | **1** | 0.943 | 0.936 | 0.603 |
| **ARF** | 0.929 | 0.929 | 0 | 0.908 | 0.886 | 0.533 |
| **CIR** | **0.937** | **0.937** | 0 | **0.995** | 0.914 | 0.677 |

^1^2.1.3: Integrity of data values as inputs in predictive modeling.

^2^2.2.2: Integrity of timestamps as creating values with data.

^3^2.4.1: Adequate data.

^4^3.1.4: Accurate measurement of data.

^5^4.1.1: Timeliness on recording data.

^6^4.1.2: Frequency on recording data.
